# The Triglyceride–Glucose Index and Its Combination with C-Reactive Protein and Anthropometric Indicators as Biomarkers for Hypertension Among Saudi Adults: A Cross-Sectional Survey

**DOI:** 10.3390/jcm15187253

**Published:** 2026-09-18

**Authors:** Azzah S. Alharbi

**Affiliations:** 1 Clinical Microbiology and Immunology Department, Faculty of Medicine, King Abdulaziz University, Jeddah 21589, Saudi Arabia; asalharbi3@kau.edu.sa; 2 Special Infectious Agents Unit, King Fahd Medical Research Center, King Abdulaziz University, Jeddah 21589, Saudi Arabia

**Keywords:** hypertension, triglyceride–glucose index, C-reactive protein, waist-to-height ratio, anthropometric indicators, inflammation, Saudi adults

## Abstract

**Background**: Although many inflammatory–metabolic and anthropometric markers are used to diagnose hypertension among adults, there is little research on how these markers can be combined to detect prevalent hypertension in the Saudi population. This study investigated the discriminatory power of an inflammation-based index combining triglyceride and glucose levels (the high-sensitivity C-reactive protein–triglyceride–glucose (hs-CRP-TyG) index), along with anthropometric indicators, to determine hypertension status in a cohort of Saudi adults. **Methods**: This study used data from a cross-sectional survey conducted in Jeddah (Saudi Arabia) involving 1299 non-diabetic adults, who were grouped based on their blood pressure state into normal BP, prehypertension, and hypertension groups. The anthropometric indicators BMI (body mass index), WC (waist circumference), WC:HT (waist-to-height ratio), WWI (weight-adjusted waist index), and CI (conicity index) were calculated. Multinomial logistic regression was performed to estimate associations between hs-CRP-TyG and hypertension and between hs-CRP-TyG + anthropometric indicators and hypertension. Interaction analyses, sex-stratified analyses, and sensitivity analyses using binomial logistic regression were performed. Receiver operating characteristic (ROC) curve analysis was performed to assess the discriminatory accuracy of the hs-CRP-TyG index and its combinations. **Results**: Baseline characteristics showed significant variations across the blood pressure categories (all *p* < 0.001). Multinomial logistic regression showed that the hs-CRP–TyG model was significantly associated with hypertension (OR = 1.487, 95% CI: 1.242–1.781; *p* = 0.001) but not with prehypertension. The WC:HT model was significantly associated with both prehypertension and hypertension and showed comparable discriminatory performance and greater clinical applicability (Nagelkerke R^2^ = 0.157). Binary logistic regression found that both hs-CRP–TyG (OR = 1.290, *p* = 0.013) and WC:HT (OR = 1.371, *p* < 0.001) were significantly associated with hypertension. The combined hs-CRP–TyG + WC:HT model had a numerically higher AUC (0.738) compared to the models with either marker alone in the overall and sex-stratified analyses. **Conclusions**: The combination of inflammation-based and anthropometric markers showed a numerically higher AUC for discriminating prevalent hypertension compared to the individual markers alone. Although these results suggest the potential utility of combining inflammation-based markers and central obesity markers, external validation is required before this approach can be integrated into clinical practice to assess prevalent hypertension status among Saudi adults.

## 1. Introduction

Hypertension is considered a leading risk factor for cardiovascular disease, stroke, chronic kidney disease, and early death globally [1,2]. Despite advances in interventions for and prevention of hypertension, its prevalence has continued to rise, especially in developing countries in Asia and the Middle East, including Saudi Arabia, due to the increasing prevalence of obesity and metabolic syndrome [3,4,5]. Thus, identifying individuals who are at an increased risk of developing hypertension is critical for successful prevention and targeting of interventions.

Hypertension results from a complex web of interactions between insulin resistance, metabolic dysfunction, vascular injury, and inflammation [6,7]. There is growing evidence that insulin resistance significantly contributes to the regulation of blood pressure via various mechanisms including sympathetic nervous system activation [8], decreased functioning of the endothelium [9], oxidative stress [10], vascular remodeling [11], and abnormal sodium handling [12,13]. The triglyceride–glucose (TyG) index has emerged as a clinical biomarker of insulin resistance. There is empirical evidence supporting its association with hypertension and cardiovascular diseases [14,15]. Many studies have concluded that TyG and its derivatives can predict hypertension incidence and adverse cardiometabolic events across various populations [16,17].

In addition to insulin resistance, inflammation also plays a pivotal role in the pathogenesis of hypertension. Accumulating evidence has implicated high-sensitivity C-reactive protein (hs-CRP), an inflammatory biomarker, in increasing arterial stiffness, impairment of endothelial function, and vascular injury [18,19], and could therefore serve as a reliable biomarker for the development and progression of hypertension. Studies have suggested that combining inflammatory biomarkers with metabolic dysfunction-related biomarkers, such as triglycerides, could better predict cardiovascular risk compared with individual biomarkers alone [6,18,20]. Indeed, some studies have shown a stronger association of composite inflammatory–metabolic indices with cardiovascular outcomes, cardiometabolic multimorbidity, and hypertensive complications compared to isolated indices [18,21].

Traditional anthropometric measures of obesity such as body mass index (BMI) are limited in their ability to accurately determine total body fat; however, recent evidence showed that waist circumference (WC), the weight-adjusted waist index (WWI), and the conicity index (CI) can more accurately predict cardiometabolic risk compared to BMI [22,23,24]. Obesity is linked with metabolic syndromes and low-grade inflammation, which jointly contribute to progression of hypertension [17,25]. Accumulating evidence suggests that hs-CRP-TyG, as a composite measure for insulin resistance and inflammation, could be used as a biomarker for cardiovascular diseases.

Despite the tremendous potential of hs-CRP-TyG, few studies have attempted to develop a framework integrating hs-CRP-TyG and anthropometric indicators to predict hypertension risk. Previous studies showed that combining the TyG index with anthropometric indicators such as WWI, waist-to-height ratio, and BMI can improve the prediction of cardiovascular diseases. Similarly, other studies have suggested combining hs-CRP-TyG-derived indicators with anthropometric and metabolic indicators to form hs-CRP-TyG-WWI, hs-CRP-TyG-BMI, and hs-CRP-TyG-WC:HT indices, which could show improved performance in the prediction of cardiovascular diseases. However, no study has attempted to systemically evaluate the discriminatory value of hs-CRP-TyG and its derivatives in combination with anthropometric measures for predicting hypertension, especially in a Saudi population.

Therefore, this study compared the performance of hs-CRP-TyG and its derivatives in combination with anthropometric measures and determined the optimal model for hypertension risk assessment. The findings from this study will facilitate the identification of prevalent hypertension, bolster its risk classification, and contribute to the development of personalized preventative interventions for individuals at high risk of hypertension.

Moreover, although the authors of [26] examined conventional cardiovascular risk factors associated with elevated blood pressure, they did not investigate the discriminatory performance of the TyG index, the combined hs-CRP–TyG index, or their interaction with anthropometric indicators for hypertension. Therefore, the present study addressed this gap by comparing the predictive performance of combinations of inflammatory–metabolic and anthropometric factors for hypertension risk to that of the individual markers.

## 2. Materials and Methods

### 2.1. Study Design and Data Source

This study utilized publicly available data from a cross-sectional survey conducted in Jeddah, Saudi Arabia, between July 2016 and February 2017 [26]. The survey implemented a stratified sampling design across the 5 geographical sectors in Jeddah, including 3 healthcare centers from each sector. The baseline cohort comprised 1299 non-diabetic adults (719 men and 580 women) aged ≥18 years, who were recruited and interviewed between 2016 and 2017; anthropometric measurements were taken, and blood samples were collected by trained field researchers using standardized tools. The study protocol was approved by the Committee on the Ethics of Human Research (CEHR) of the Faculty of Medicine, King Abdulaziz University, Jeddah (Reference No. 338–10), and was carried out in line with the principles of the Declaration of Helsinki. Written informed consent was obtained from all participants after the study objectives and procedures were explained. Further information about the complete study design and sampling procedure is available in [26,27].

**Figure 1:**
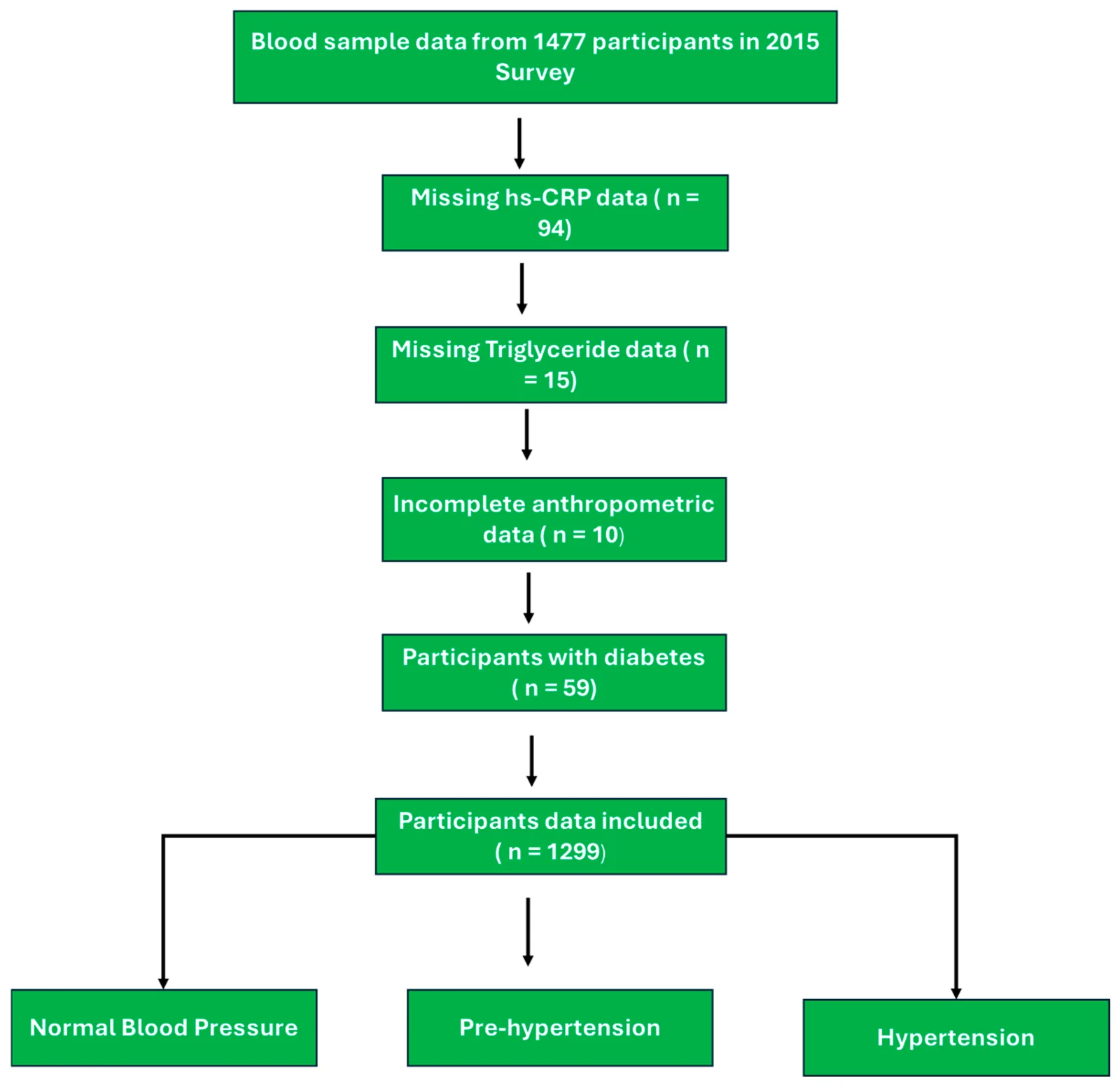
Flowchart displaying the participant selection/exclusion process in this study.

From an initial pool of 1477 participants with available blood samples, we applied the following exclusion criteria: missing hs-CRP (*n* = 94) or triglyceride data (*n* = 15), or incomplete anthropometric (*n* = 10) or diabetic data (*n* = 59). After excluding these participants, a total of 1299 participants (719 men and 580 women) were included in the final analysis. Thus, the present study used a defined subset of the original cohort that was selected according to the requirements of the current research objectives rather than reproducing the analytical approach of the original publication. The participants were grouped into normal blood pressure, prehypertension, and hypertension groups. A flowchart depicting the participant selection process is shown in Figure 1.

### 2.2. Biochemical Measurements

Fasting venous blood samples were collected from all participants after an overnight fast and analyzed at an accredited laboratory. Plasma glucose, hs-CRP, total cholesterol (TC), high-density lipoprotein cholesterol (HDL-C), and triglycerides (TG) were measured using spectrophotometric methods and an Architect c8000 auto-analyzer (Abbott Laboratories, Abbott Park, IL, USA) as described in [26]. Low-density lipoprotein cholesterol (LDL-C) concentrations were calculated using the Friedewald equation [28]. Glycated hemoglobin (HbA1c) was measured by high-performance liquid chromatography (HPLC) using an automated G8 HbA1c analyzer (TOSOH Corporation, Tokyo, Japan). Fasting plasma glucose, triglyceride, and hs-CRP measurements were used to calculate the TyG and hs-CRP–TyG indices. Lipid profile parameters were used for descriptive analyses and participant characterization.

### 2.3. Classification of Outcome Variable

The outcome variable was hypertension, which was defined according to the Seventh Report of the Joint National Committee on Prevention, Detection, Evaluation, and Treatment of High Blood Pressure (JNC7) [29]. Normal blood pressure was defined as a systolic blood pressure (SBP) < 120 mmHg and diastolic blood pressure (DBP) < 80 mmHg. Prehypertension was defined as an SBP of 120–139 mmHg and/or DBP of 80–89 mmHg. Hypertension was defined as an SBP ≥ 140 mmHg, DBP ≥ 90 mmHg, or current use of antihypertensive medication. The JNC7 classification of ‘prehypertension’ was retained for consistency with the blood-pressure categories used in the original analysis of the dataset used in this study, although contemporary guidelines may use different terminologies such as ‘Stage 1 hypertension’, which is used by the American College of Cardiology and American Heart Association (ACC/AHA).

### 2.4. Calculation of hs-CRP-TyG and Its Derivative Indices

All indicators were calculated using hs-CRP, triglycerides, fasting blood glucose, triglyceride, weight, WC, and height (HT). The formulas are as follows: BMI [30] = Weight (kg)/(Height [m])^2^; TyG [31,32] = Ln [fasting triglycerides (mg/dL) × fasting glucose (mg/dL)]/2; hs-CRP-TyG [30] = 0.412 × ln(hs-CRP [mg/L]) + ln(TG [mg/dL] × FPG [mg/dL])/2; WC:HT [30] = WC (m)/Height (m); CI [33] = $WC(m)/0.109\times \sqrt{\frac{weight(kg)}{height(m)}}$; WWI [33] = WC(m)/$\sqrt{weight(kg)}$. These variables were also examined for their associations with blood pressure categories and their discriminatory ability for hypertension.

### 2.5. Statistical Analysis

Statistical analyses were performed using IBM SPSS Statistics version 29.0 (IBM Corp., Armonk, NY, USA). Continuous variables are presented as the mean ± standard deviation (SD), and categorical variables are presented as frequencies and percentages. Comparisons between males and females were conducted using the Mann–Whitney U test or independent samples *t*-test. The chi-square test was applied to compare categorical variables. All continuous discriminators were standardized as z-scores before regression analyses; therefore, odds ratios (ORs) represent the effect per 1 SD increase. Statistical significance was defined as a two-tailed *p* < 0.05.

Differences across blood pressure categories (non-hypertensive, prehypertensive, and hypertensive) were assessed using one-way analysis of variance (ANOVA) with Tukey’s post hoc test for continuous variables and chi-square tests with Bonferroni correction for categorical variables. Multicollinearity was assessed before regression modeling. Associations between hs-CRP–TyG, anthropometric indices, and blood pressure categories were examined using multinomial logistic regression, with the normal blood pressure group as the reference group. Separate models were fitted for each anthropometric indicator to minimize collinearity and were analyzed before and after adjustment for age and sex. Model performance was evaluated using −2 Log Likelihood, Nagelkerke R^2^, and Likelihood Ratio chi-square statistics.

The interaction between hs-CRP–TyG and WC:HT was evaluated by adding a multiplicative interaction term to the regression model. Because the strongest associations were observed for hypertension, binary logistic regression was subsequently performed after excluding prehypertensive participants, with hypertension coded as 1 and normal blood pressure as 0. Model fit was assessed using the Hosmer–Lemeshow goodness-of-fit test and Nagelkerke R^2^. Sex-stratified analyses were also conducted.

The discriminatory values of the combined hs-CRP–TyG and WC:HT model were determined through multivariable logistic regression. Subsequently, receiver operating characteristic (ROC) curve analyses were performed to evaluate the discriminatory performance of hs-CRP–TyG, WC:HT, and their combination for hypertension. The area under the curve (AUC) with 95% confidence intervals was used to determine the discriminatory accuracies of all models. The maximum Youden index was employed to determine the optimal cut-off values, followed by calculation of the corresponding sensitivity and specificity values. Exclusion criterion based on markedly elevated hs-CRP concentrations (>10 mg/L) was applied, and sensitivity analyses excluding such values were performed.

## 3. Results

### 3.1. Baseline Characteristics According to Blood Pressure Status

Table 1 presents the baseline characteristics of the participants according to blood pressure status (non-hypertensive, prehypertensive, and hypertensive groups). Significant differences were observed across all measured variables (all *p* < 0.001).

The participants with hypertension were older than those with normal blood pressure or prehypertension, with the mean age increasing progressively across blood pressure categories (30.88 ± 10.23 years in the non-hypertensive group, 33.34 ± 10.16 years in the prehypertensive group, and 40.20 ± 14.36 years in the hypertensive group). The anthropometric measures also increased with worsening blood pressure status. Individuals with hypertension had higher BMI, WC, WC:HT ratio, conicity index, and weight-adjusted waist index values compared with non-hypertensive participants.

Similarly, metabolic and inflammatory markers increased across blood pressure categories. The highest mean TyG and hs-CRP–TyG values were observed in the hypertensive group, suggesting greater metabolic dysregulation and inflammatory burden. As expected, systolic and diastolic blood pressure values were significantly higher in the prehypertensive and hypertensive groups compared to the normal blood pressure group.

The sex distribution also differed significantly across groups (*p* < 0.001), indicating potential sex-related differences in blood pressure status distribution. Overall, these findings suggest that increasing blood pressure status is associated with adverse anthropometric, metabolic, and inflammatory profiles.

### 3.2. Association of hs-CRP–TyG Combined with Anthropometric Indices and Blood Pressure Categories

Table 2 summarizes the multinomial logistic regression analyses examining the associations between hs-CRP–TyG combined with different anthropometric indices and blood pressure categories.

hs-CRP–TyG alone was not significantly associated with prehypertension in either the unadjusted or adjusted analyses. However, hs-CRP–TyG demonstrated significant associations with hypertension, with adjusted analyses showing a 48.7% increase in hypertension odds (OR = 1.487, *p* = 0.001), suggesting stronger involvement in established hypertension rather than earlier blood pressure abnormalities.

Most anthropometric indices demonstrated significant associations with both prehypertension and hypertension when combined with hs-CRP–TyG. Among these, WC, WC:HT, WWI, and conicity index remained independently associated after adjustment for age and sex. For hypertension, the effect estimates for the different anthropometric indices were broadly comparable, with overlapping confidence intervals indicating similar discriminatory contributions.

The combined hs-CRP–TyG + WC:HT model showed that WC:HT was significantly associated with increased odds of prehypertension (OR = 1.508, *p* = 0.011) and hypertension (OR = 1.399, *p* = 0.001); however, hs-CRP–TyG was found to be independently associated with hypertension. No significant association was found between hs-CRP–TyG and prehypertension status.

### 3.3. Comparison of Performance Models Integrating Different Anthropometric Indicators and Their Interaction Effects

The performance comparison of the hs-CRP–TyG models integrating different anthropometric metrics is presented in Table 3. Both the unadjusted and adjusted analyses found statistically significant fits for all models (all *p* < 0.001). After adjusting the models for age and sex, substantial improvements in model performance were observed, as reflected by the lower −2 Log Likelihood values and increased Nagelkerke R^2^ values.

The adjusted models integrating central obesity indices (WC, WC:HT, WWI, and conicity index) exhibited comparable explanatory ability for the outcome (Nagelkerke R^2^ = 0.157 to 0.165). For example, the Nagelkerke R^2^ values for the hs-CRP–TyG + WC and WC:HT models were 0.165 and 0.157, respectively, reflecting the statistically comparable discriminatory abilities of both models that use central obesity indices. The latter model was selected for subsequent analyses due to its simpler clinical interpretation, widespread use in previous studies, and greater utility in public health settings.

Interaction analyses examining the synergistic effects of the hs-CRP–TyG and WC:HT models in discriminating between blood pressure categories are presented in Table 4. Significant interaction effects were observed in both unadjusted (OR = 0.628, *p* = 0.004) and adjusted models (OR = 0.660, *p* = 0.014) for prehypertension. These results suggest that WC:HT may affect the relationship between hs-CRP–TyG and prehypertension status.

In contrast, after adjustment, no statistically significant interaction effect was observed for hypertension (OR = 1.020, *p* = 0.837). These results indicate that hs-CRP–TyG and WC:HT each contribute independently, but they do not perform synergistically in determining hypertension status.

Model diagnostics were performed on the adjusted interaction model using the Nagelkerke R^2^ as a goodness-of-fit measure. The Nagelkerke R^2^ value was 0.164, indicating that the model explained 16.4% of the variance in blood pressure categories. These data further supported the modulating effect of the interaction.

### 3.4. Association of hs-CRP–TyG and WC:HT with Hypertension: Binary Logistic Regression Analysis

Binary logistic regression analysis was performed to evaluate the associations between hs-CRP–TyG, WC:HT, and hypertension after excluding individuals with prehypertension (Table 5). In the adjusted model, both hs-CRP–TyG and WC:HT were significantly associated with increased odds of hypertension. Specifically, each 1 SD unit increase in hs-CRP–TyG was associated with a 29.0% increase in the odds of hypertension (OR = 1.290, *p* = 0.013), while WC:HT was associated with a 37.1% increase in the odds of hypertension (OR = 1.371, *p* < 0.001).

Age was also identified as a significant discriminator, with increasing age associated with higher odds of hypertension (OR = 1.049, *p* < 0.001). Sex showed borderline statistical significance (OR = 1.394, *p* = 0.052). Model diagnostics suggested acceptable fit, with a Nagelkerke R^2^ value of 0.171, indicating that approximately 17.1% of the variation in hypertension status was explained by the model. Furthermore, the non-significant Hosmer–Lemeshow test result (χ^2^ = 8.952, df = 8, *p* = 0.346) indicated good model calibration.

The sex-stratified analyses are presented in Table 6. Among females, hs-CRP–TyG was not significantly associated with hypertension risk (OR = 1.135, *p* = 0.443). However, WC:HT remained significantly associated with hypertension, with a 34.1% increase in odds observed per 1 SD unit increase (OR = 1.341, *p* = 0.046). Age also remained an independent discriminator among females (OR = 1.050, *p* < 0.001).

In contrast, among males, both hs-CRP–TyG and WC:HT were significantly associated with hypertension. hs-CRP–TyG increased the odds of hypertension by 40.5% (OR = 1.405, *p* = 0.010), while WC:HT was associated with a 41.9% increase in odds (OR = 1.419, *p* = 0.006). Age remained a significant discriminator in males as well (OR = 1.050, *p* < 0.001).

The model performance statistics indicated acceptable fit for both sex-specific models. Among females, the model demonstrated a Nagelkerke R^2^ of 0.168 and a non-significant Hosmer–Lemeshow test result (χ^2^ = 10.433, df = 8, *p* = 0.236), while the male model showed a slightly higher explanatory power (Nagelkerke R^2^ = 0.178) and adequate calibration (χ^2^ = 6.650, df = 8, *p* = 0.574). Overall, these findings suggest sex-specific differences in the relationship between hs-CRP–TyG and hypertension, whereas WC:HT and age remained consistent discriminators in both sexes.

### 3.5. Discriminatory Performance of hs-CRP–TyG and WC:HT for Hypertension

The hs-CRP–TyG model’s discriminatory ability, sensitivity, and specificity was analyzed (Table 7; Figure S1). The results showed that the model had a moderate discriminatory ability for distinguishing hypertensive from non-hypertensive individuals, with a sensitivity of 74.4% and specificity of 53.1%. These data indicate that the model will incorrectly classify a proportion of non-hypertensive individuals as hypertensive. Therefore, the model’s discriminatory performance should be interpreted cautiously, particularly when distinguishing hypertensive from non-hypertensive individuals.

Compared with the hs-CRP-TyG model, the WC:HT model showed a numerically higher AUC (0.70), with a sensitivity of 68.2%, specificity of 67.0%, and Youden index of 0.35 for discriminating between hypertensive and non-hypertensive individuals. The combined hs-CRP–TyG + WC:HT model showed a numerically higher AUC (0.73), with a sensitivity of 65.2%, specificity of 74.5%, and Youden index of 0.39.

### 3.6. Sex-Specific Discriminatory Performance of hs-CRP–TyG and WC:HT for Hypertension

The sex-specific discriminatory performance of the WC:HT, hs-CRP–TyG, and combined hs-CRP–TyG + WC:HT models was assessed (Table 8; Figures S2–S4). All three models showed significant discriminatory performance (all *p* < 0.001) for both genders in terms of discriminating between hypertensive and non-hypertensive individuals.

However, different models showed varying performance in terms of discrimination of hypertension in males and females. For example, among females, the WC:HT model showed an AUC of 0.714, sensitivity of 67.8%, specificity of 68.2%, and Youden index of 0.36 compared with values of 0.65, 61%, 67%, and 0.28 for the hs-CRP-TyG model. The combined hs-CRP–TyG + WC:HT model exhibited a numerically higher AUC (0.736), with a sensitivity of 63%, specificity of 79.5%, and Youden index of 0.42.

Similarly, among males, the combined hs-CRP–TyG + WC:HT model showed a numerically higher AUC (0.739), with a sensitivity of 65.8%, specificity of 73%, and Youden index of 0.388, indicating relatively good discriminatory performance for hypertension. The WC:HT model showed a numerically higher AUC (0.695), with a sensitivity of 70.2%, specificity of 65.1%, and Youden index of 0.35 compared to the values of 0.666, 76.0%, 51.5%, and 0.27 for the hs-CRP–TyG model.

## 4. Discussion

This study examined the associations of inflammatory–metabolic and anthropometric indicators with blood pressure categories and evaluated the discriminatory performance of hs-CRP–TyG composite models for hypertension. Several key points emerged from the results. Firstly, anthropometric, metabolic, and inflammatory factors progressively increased across blood pressure categories, reflecting increasing cardiometabolic burden from prehypertension to hypertension status. Secondly, hs-CRP-TyG, even after adjustment for age and sex, showed significant associations with hypertension but not with prehypertension, which indicates that inflammatory and metabolic disturbances may act at a later stage of blood pressure dysregulation. Thirdly, anthropometric indicators of central adiposity were significantly associated with both prehypertension and hypertension, highlighting the significance of central obesity throughout the progression of blood pressure dysregulation. Finally, the model combining hs-CRP-TyG and anthropometric indicators showed a numerically higher AUC than the hs-CRP-TyG model, indicating relatively good discriminatory performance for hypertension. Among the anthropometric indicators, the waist-to-height ratio may have greater potential clinical applicability because of its simplicity and discriminatory performance that is numerically comparable to that of hs-CRP-TyG. These findings suggest that the hs-CRP-TyG + WC:HT combination may have potential clinical utility for discriminating hypertension.

Anthropometric and metabolic parameters showed an increase from normotensive subjects to hypertensive patients, suggesting that both metabolic disturbances and adiposity burden worsen with increasing blood pressure. These findings are consistent with previously published literature that demonstrated that obesity-related anthropometric markers and metabolic disturbances worsen from a normotensive to hypertensive state [14,22]. Additionally, studies comparing multiple obesity indicators have demonstrated that central adiposity indicators are more strongly correlated with hypertension than general obesity indicators [34,35]. Therefore, these data suggest that adiposity-driven metabolic disturbances begin early in the development of blood pressure dysregulation and continue to be a major contributor to vascular dysfunction as it progresses.

A novel finding of this study was that hs-CRP-TyG retained independent associations with hypertension, but not with prehypertension, after adjusting for confounders; this aligns with previous research demonstrating a relationship between TyG-related indices and hypertension risk [15,36]. Both prospective and cross-sectional studies have found that elevated TyG correlates with insulin resistance and predicts adverse cardiovascular outcomes, including incident hypertension [16,17]. Our data demonstrating the potential of combining inflammation indicators, such as CRP, to detect hypertension but not early blood pressure abnormalities has a biologically plausible explanation. Insulin resistance and inflammation lead to sympathetic nervous system activation [8], endothelial dysfunction [9], oxidative stress [10], vascular remodeling [11], and altered sodium handling [12]; these dysfunctions become more pronounced during prolonged periods of elevated blood pressure [37].

Additionally, our findings demonstrate that inflammatory mechanisms can potentiate the deleterious cardiovascular consequences of metabolic disturbances. Other researchers have demonstrated that combining TyG with inflammatory biomarkers enhances prediction and discrimination accuracy for cardiovascular events compared to using only metabolic or inflammatory indicators [14,18,20]. More recently, other researchers demonstrated that inflammatory mediators partially explain the relationship between TyG-derived indices and adverse cardiovascular events, further indicating that inflammation is a critical intermediate mechanism linking metabolic disturbance and vascular disease [18,38,39]. However, our data slightly contradict those of studies demonstrating stronger associations between TyG derivatives and prehypertension or the transition from prehypertension to hypertension [40,41]. Such differences could be due to differences in study population, ethnicity, study design, or the addition of an inflammatory component into hs-CRP-TyG.

A strength of this study was that it simultaneously assessed several different anthropometric indicators combined with hs-CRP-TyG. All five anthropometric indicators (BMI, waist circumference, WC:HT, WWI, and conicity index) showed significant associations with hypertension, with relatively minor differences in discriminatory performance. This finding is consistent with prior studies demonstrating similar discriminatory abilities among various central obesity indicators [23,35]. Prior studies have likewise found that combining obesity-related indicators and TyG improves discrimination of prevalent hypertension status and/or cardiovascular outcomes compared to using individualized markers [25,40]. Other studies assessing TyG-related composite markers have demonstrated enhanced discriminatory capacity for hypertension [42,43], multimorbidity [43], and cardiovascular disease [30], which supports our finding that multi-dimensional indices provide better representations of overall cardiometabolic risk.

Although multiple anthropometric indicators showed comparable discriminatory performance, as observed in other studies [22,24] the WC:HT ratio was selected for the final model due to its simplicity, low cost, and wider utility within clinical and public healthcare settings. Previous studies also concluded that waist-based measures are more practical and effective for screening individuals at increased risk of cardiometabolic disorders and hypertension [22,30,31]. The numerically higher AUC observed for the combined hs-CRP-TyG + WC:HT model suggests that combining inflammatory–metabolic and anthropometric measures may have better utility for discriminating hypertension compared with either type of marker alone. In the interaction analyses investigating the synergistic effects of metabolic dysfunction and central adiposity on different stages of hypertension, a significant interaction between hs-CRP–TyG and WC:HT was observed in prehypertensive individuals but not hypertensive subjects after adjustment for age and sex. These results stand in contrast with previous studies that demonstrated synergistic associations between inflammatory–metabolic biomarkers and adiposity in risk stratification for cardiovascular diseases [44]. The significant hs-CRP–TyG × WC:HT interaction for prehypertension but not hypertension indicates that the association of inflammatory–metabolic status and central adiposity may differ in different blood pressure categories; nevertheless, a cautious approach should be adopted while interpreting this statistical interaction as evidence of a temporal, causal, or biological interaction within the cross-sectional design of this study. The absence of a significant interaction for hypertension in this study may reflect differences in the relative contribution of these factors across blood pressure categories in the Saudi population.

The sex-specific analyses revealed that hs-CRP-TyG was significantly associated with hypertension prevalence in males but not in females, whereas WC:HT retained significant discriminatory value for both males and females. However, these subgroup-specific patterns should not be interpreted as evidence of effect modification by sex; this should be tested in future prospective studies through hs-CRP–TyG × sex interaction analysis.

The combined model discriminatory analyses demonstrated that hs-CRP-TyG + WC:HT consistently showed a numerically higher AUC than hs-CRP-TyG. Although the differences in AUC values were small, the combined model showed a relatively high discrimination capability with a more desirable trade-off between sensitivity and specificity. Collectively, such findings are supportive of the emerging evidence indicating that multi-dimensional biomarkers integrating inflammatory, insulin-resistance, and obesity indicators may offer enhanced discriminatory capabilities for cardiometabolic disease [21,23,45,46]. As both hs-CRP-TyG and WC:HT represent low-cost and easily measurable parameters, their combined use could serve as a clinically viable option for identifying individuals at increased risk for developing hypertension in resource-poor environments such as Saudi Arabia.

Previous studies have often evaluated anthropometric or TyG-derived indices separately for identification of hypertension [46,47]. This study contributes to the existing research by integrating inflammatory–metabolic and anthropometric indicators (hs-CRP–TyG + WC:HT) to identify prevalent hypertension. The comparison of multiple anthropometric indicators identified WC:HT as a simple and practical marker with good discriminatory performance that is comparable to the other indices evaluated. Sex-stratified and blood-pressure-category analyses provided additional exploratory evidence of the consistency of these associations across participant subgroups.

This study had limitations in addition to its strengths and contributions. Specifically, the cross-sectional nature of this study does not allow for causal inferences nor permit the examination of time-dependent relationships. Additionally, residual confounding from other factors—including lifestyle habits, medication usage, levels of physical activity, smoking status, diet, and other environmental factors—may have affected the results. Misclassification bias may have occurred as the blood pressure classification was based on a limited number of blood pressure measurements. Moreover, the study was geographically restricted to one region (Jeddah) of Saudi Arabia, which may limit the generalizability of the findings to the wider Saudi population. In addition, potential selection bias associated with participant recruitment and the eligibility criteria cannot be excluded.

Importantly, the cross-sectional design precludes the assessment of temporality and incident hypertension. Therefore, although the ROC analyses quantify the performance of all three models (hs-CRP–TyG, WC:HT, and their combination) in discriminating prevalent hypertension, they should not be interpreted as predictive models for hypertension. Prospective longitudinal studies could corroborate the temporal and prognostic utility of these markers in predicting incident hypertension.

Additionally, while combined models did improve discriminatory accuracy, the overall discriminative performance of the models remained moderate; incorporating other biological, genetic, and environmental factors not tested in the current study could improve discriminatory performance. Furthermore, no internal validation was performed on the models; therefore, the reported discriminatory performance metrics (AUC, sensitivity, and specificity) may be optimistic and should be interpreted cautiously.

## 5. Conclusions

The hs-CRP–TyG index and anthropometric indicators were independently associated with prevalent hypertension, whereas central obesity measures, such as WC:HT, were significantly associated with both prehypertension and hypertension. The combined hs-CRP–TyG + WC:HT model showed a numerically higher AUC than the individual markers for discriminating prevalent hypertension. These findings suggest that integrating central adiposity and inflammatory–metabolic indicators could improve the prediction of prevalent hypertension risk. Nevertheless, given the cross-sectional design of the study, the findings should not be interpreted as established causal relationships or a method for predicting future hypertension. Prospective longitudinal studies are needed to determine whether hs-CRP–TyG and WC:HT, individually or in combination, can predict incident hypertension.

## Figures and Tables

**Table 1:** Baseline characteristics of study population.

Variable	Non-Hypertensive (Mean ±SD) (N = 622)	Prehypertensive (Mean ± SD) (N = 480)	Hypertensive (Mean ± SD) (N = 197)	*p*-Value
Age	30.88 ± 10.23	33.34 ± 10.16	40.20 ± 14.36 b	<0.001
Male (%)	269 (43.25%)	338 (70.42%) a	112 (56.85%)	<0.001
Female (%)	353 (56.75%)	142 (29.58%) a	85 (43.15%)	<0.001
BMI (kg/m^2^)	26.7 ± 5.76	28.74 ± 4.9 a	30.7 ± 6.7 b	<0.001
WC (cm)	90.8 ± 15.5	99.79 ± 12.8 a	102 ± 17.04 b	<0.001
WC:HT	0.55 ± 0.09	0.59 ± 0.07 a	0.61 ± 0.1 b	<0.001
C index	2.2 ± 0.27	2.35 ± 0.21 a	2.38 ± 0.29 b	<0.001
WWI	21.1 ± 2.66	22.6 ± 2.09 a	23 ± 2.87 b	<0.001
TyG	8.09 ± 0.71	8.22 ± 0.87	8.54 ± 0.68 b	<0.001
hs-CRP-TyG	4.61 ± 0.67	4.7 ± 0.71	4.99 ± 0.63 b	<0.001
SBP (mmHg)	113.66 ± 10.84	127.51 ± 5.65 a	136.75 ± 19.35 b	<0.001
DBP (mmHg)	69.79 ± 9.1	85.89 ± 1.39 a	87.59 ± 11.68 b	<0.001

Note: Data reported as mean (± SD) for continuous variables and number (%) for categorical variables. *p*-values from ANOVA with Tukey’s post hoc test for continuous variables and chi-square test with Bonferroni correction for categorical variables for differences between the three groups. Differences are significant if *p* < 0.05. a: *p* < 0.05 between normal and prehypertensive groups, b: *p* < 0.05 between normal and hypertensive groups. Abbreviations: BMI, body mass index; WC, waist circumference; WC:HT, waist-to-height ratio; C index, conicity index; WWI, weight-adjusted waist index; TyG, triglyceride–glucose index; hs-CRP-TyG, high-sensitivity C-reactive protein–triglyceride–glucose index; SBP, systolic blood pressure; DBP, diastolic blood pressure.

**Table 2:** Association between hs-CRP–TyG combined with different anthropometric indices and hypertension.

Model	Variable	Prehypertension						Hypertension					
		Unadjusted			Adjusted for Age and Sex			Unadjusted			Adjusted for Age and Sex		
		OR	95% CI	*p*-Value	OR	95% CI	*p*	OR	95% CI	*p*	OR	95% CI	*p*-Value
hs-CRP-TyG		1.175	0.893–1.546	0.249	1.066	0.802–1.418	0.659	1.841	1.552–2.183	<0.001	1.487	1.242–1.781	0.001
hs-CRP-TyG-BMI	BMI	1.362	1.00–1.855	0.05	1.304	0.959–1.773	0.09	1.547	1.30–1.84	<0.001	1.462	1.223–1.748	<0.001
	hs-CRP-TyG	1.004	0.732–1.376	0.981	0.952	0.699–1.296	0.754	1.443	1.187–1.754	<0.001	1.242	1.019–1.512	0.032
hs-CRP-TyG-WC	WC	1.875	1.398–2.516	<0.001	1.599	1.164–2.197	0.004	1.738	1.453–2.079	<0.001	1.536	1.266–1.864	<0.001
	hs-CRP-TyG	0.858	0.633–1.161	0.321	0.879	0.648–1.194	0.409	1.396	1.148–1.698	<0.001	1.243	1.019–1.517	0.032
hs-CRP-TyG-WC:HT	WC:HT	1.599	1.182–2.163	0.002	1.508	1.097–2.075	0.011	1.655	1.385–1.978	<0.001	1.399	1.159–1.689	0.001
	hs-CRP-TyG	0.915	0.669–1.252	0.578	0.888	0.66–1.22	0.492	1.411	1.157–1.720	<0.001	1.288	1.056–1.572	0.013
hs-CRP-TyG-WWI	WWI	1.82	1.353–2.448	<0.001	1.596	1.155–2.204	0.005	1.688	1.410–2.021	<0.001	1.414	1.167–1.714	<0.001
	hs-CRP-TyG	0.880	0.651–1.190	0.408	0.894	0.661–1.210	0.469	1.435	1.182–1.742	<0.001	1.304	1.072–1.586	0.008
hs-CRP-TyG-CI	CI	1.878	1.399–2.520	<0.001	1.618	1.169–2.241	0.004	1.679	1.404–2.008	<0.001	1.432	1.179–1.738	<0.001
	CRP-TyG	0.880	0.653–1.185	0.399	0.897	0.663–1.212	0.477	1.456	1.202–1.764	<0.001	1.306	1.075–1.588	0.007

Abbreviations: hs-CRP: high-sensitivity C-reactive protein; TyG: triglyceride–glucose index; BMI: body mass index; WC: waist circumference; HT: height; WWI: weight-adjusted waist index; CI: conicity index.

**Table 3:** Performance comparison of hs-CRP–TyG models integrating different anthropometric indices in discriminating blood pressure categories.

Model	Unadjusted					Adjusted				
	−2 Log Likelihood	Nagelkerke R^2^	Likelihood Ratio (χ^2^)	df	*p*	−2 Log Likelihood	Nagelkerke R^2^	Likelihood Ratio (χ^2^)	df	*p*
hs-CRP-TyG	1530.094	0.058	55.039	2	<0.001	1451.974	0.137	133.159	6	<0.001
hs-CRP-TyG-BMI	1503.971	0.085	81.161	4	<0.001	1433.375	0.155	151.758	8	<0.001
hs-CRP-TyG-WC	1447.743	0.110	104.031	4	<0.001	1393.074	0.165	158.701	8	<0.001
hs-CRP-TyG-WC:HT	1460.163	0.098	91.611	4	<0.001	1400.982	0.157	150.792	8	<0.001
hs-CRP-TyG-WWI	1452.582	0.105	99.193	4	<0.001	1399.146	0.159	152.629	8	<0.001
Hs-CRP-TyG-CI	1451.079	0.107	100.695	4	<0.001	1389.219	0.160	153.555	8	<0.001

Abbreviations: hs-CRP: high-sensitivity C-reactive protein; TyG: triglyceride–glucose index; BMI: body mass index; WC: waist circumference; HT: height; WWI: weight-adjusted waist index; CI: conicity index.

**Table 4:** Performance of the hs-CRP–TyG and WC:HT models and their interaction (CRP-TyG*WC:HT).

Variable	Prehypertension vs. Normal						Hypertension vs. Normal					
	Unadjusted			Adjusted for Age and Sex			Unadjusted			Adjusted for Age and Sex		
	OR	95% CI	*p*-Value	OR	95% CI	*p*-Value	OR	95% CI	*p*	OR	95% CI	*p*-Value
hs-CRP-TyG	1.00	0.728–1.375	0.998	0.984	0.717–1.351	0.922	1.417	1.159–1.731	<0.001	1.277	1.045–1.562	0.017
WC:HT	1.704	1.245–2.332	<0.001	1.631	1.166–2.282	0.004	1.719	1.407–2.101	<0.001	1.373	1.108–1.703	0.004
hs-CRP-TyG*WC:HT	0.628	0.456–0.864	0.004	0.66	0.483–0.922	0.014	0.907	0.757–1.087	0.292	1.020	0.846–1.229	0.837

Abbreviations: hs-CRP: high-sensitivity C-reactive protein; TyG: triglyceride–glucose index; WC: waist circumference; HT: height. Unadjusted model: −2 Log Likelihood = 1450.223; Nagelkerke R^2^ = 0.108; Likelihood Ratio χ^2^ = 101.552; df = 6; *p* <0.001. Adjusted model: −2 Log Likelihood = 1393.940; Nagelkerke R^2^ = 0.164; Likelihood Ratio χ^2^ = 157.8; df = 10; *p* <0.001.

**Table 5:** Binary logistic regression of hs-CRP–TyG and WC:HT for hypertension vs. normal blood pressure.

Variable	OR	95% CI	*p*-Value
hs-CRP-TyG	1.290	1.056–1.577	0.013
WC:HT	1.371	1.137–1.654	<0.001
Age	1.049	1.035–1.064	<0.001
Sex	1.394	0.997–1.950	0.052

Abbreviations: hs-CRP: high-sensitivity C-reactive protein; TyG: triglyceride–glucose index; WC: waist circumference; HT: height. −2 Log Likelihood = 959.396; Nagelkerke R^2^ = 0.171; Hosmer–Lemeshow test: chi-square χ^2^ = 8.952, df = 8. *p* = 0.346. **Note**: Outcome defined as hypertension (1) versus normal blood pressure (0); prehypertensive individuals were excluded.

**Table 6:** Binary logistic regression of hs-CRP–TyG and WC:HT for hypertension vs. normal blood pressure in males and females.

Variable	Female			Male		
	OR	95% CI	*p*-Value	OR	95% CI	*p*-Value
hs-CRP-TyG	1.135	0.822–1.567	0.443	1.405	1.084–1.822	0.01
WC:HT	1.341	1.006–1.788	0.046	1.419	1.106–1.822	0.006
Age	1.05	1.028–1.073	<0.001	1.05	1.03–1.069	<0.001

Abbreviations: hs-CRP: high-sensitivity C-reactive protein; TyG: triglyceride–glucose index; WC: waist circumference; HT: height. Females: −2 Log Likelihood = 403.547; Nagelkerke R^2^ = 0.168; Hosmer–Lemeshow test: chi-square = 10.433, df = 8, *p* <0.236. Males: −2 Log Likelihood = 554.0; Nagelkerke R^2^ = 0.178; Hosmer–Lemeshow test: chi-square = 6.65, df = 8, *p* <0.574.

**Table 7:** Discriminatory performance of hs-CRP–TyG and WC:HT for hypertension.

Model	AUC	95% CI	*p*-Value	Std. Error	Optimal Cutoff	Sensitivity	Specificity	Youden Index
hs-CRP-TyG	0.663	0.623–0.703	<0.001	0.02	0.019	74.4%	53.1%	0.275
WC:HT	0.704	0.666–0.743	<0.001	0.02	0.156	68.2%	67%	0.352
hs-CRP-TyG + WC:HT	0.738	0.698–0.777	<0.001	0.02	0.177	65.2%	74.5%	0.397

Abbreviations: hs-CRP: high-sensitivity C-reactive protein; TyG: triglyceride–glucose index; WC: waist circumference; HT: height.

**Table 8:** Discriminatory performance of hs-CRP–TyG and WC:HT for hypertension in females and males.

**Model**	**Females**							
	**AUC**	**95% CI**	***p*-Value**	**Std. Error**	**Optimal Cutoff**	**Sensitivity**	**Specificity**	**Youden Index**
hs-CRP-TyG	0.658	0.595–0.722	<0.001	0.032	0.410	61%	67.4%	0.284
WC:HT	0.714	0.657–0.772	<0.001	0.029	0.186	67.8%	68.2%	0.360
hs-CRP-TyG + WC:HT	0.736	0.674–0.799	<0.001	0.032	0.185	63.0%	79.5%	0.425
**Model**	**Males**							
	**AUC**	**95% CI**	***p*-value**	**Std. error**	**Optimal cutoff**	**Sensitivity**	**Specificity**	**Youden index**
hs-CRP-TyG	0.666	0.614–0.717	<0.001	0.026	0.005	76%	51.5%	0.275
WC:HT	0.695	0.643- 0.748	<0.001	0.027	0.119	70.2%	65.1	0.353
hs-CRP-TyG + WC:HT	0.739	0.687–0.791	<0.001	0.027	0.182	65.8%	73%	0.388

Abbreviations: hs-CRP: high-sensitivity C-reactive protein; TyG: triglyceride–glucose index; WC: waist circumference; HT: height.

## Data Availability

The datasets used in this study can be accessed at doi: 10.1371/journal.pone.0246568).

## Supplementary Materials

The following supporting information can be downloaded at: https://www.mdpi.com/article/10.3390/jcm15187253/s1, Figure S1. Receiver operating characteristic (ROC) curves for hs-CRP–TyG, WC: HT, and their combined model in predicting hypertension; Figure S2. Receiver operating characteristic (ROC) curves for hs-CRP–TyG in male and female categories for prediction of hypertension; Figure S3. Receiver operating characteristic (ROC) curves for WC: HT model for prediction of hypertension by sex; Figure S4. Receiver operating characteristic (ROC) curves for hs-CRP–TyG +WC: HT model for prediction of hypertension by sex.

## Abbreviations

ANOVA	Analysis of variance
AUC	Area Under the Receiver Operating Characteristic Curve
BMI	Body mass index
BP	Blood pressure
CI	Conicity index
CRP	C-reactive protein
hs-CRP–TyG	C-reactive protein–triglyceride–glucose index
DBP	Diastolic blood pressure
HDL-C	High-density lipoprotein cholesterol
hs-CRP	High-sensitivity C-reactive protein
LDL-C	Low-density lipoprotein cholesterol
OR	Odds ratio
ROC	Receiver operating characteristic
SBP	Systolic blood pressure
SD	Standard deviation
TC	Total cholesterol
TG	Triglycerides
TyG	Triglyceride–glucose index
WC	Waist circumference
WC:HT	Waist-to-height ratio
WWI	Weight-adjusted waist index
HbA1c	Hemoglobin A1c
