# Supplementary material for: The Triglyceride–Glucose Index and Its Combination with C-Reactive Protein and Anthropometric Indicators as Biomarkers for Hypertension Among Saudi Adults: A Cross-Sectional Survey
Source: J Clin Med. 2026 Sep 18;15(18):7253. doi: 10.3390/jcm15187253 (PMC13607200; doi:10.3390/jcm15187253)
Supplement: Supplementary file 1 [file jcm-15-07253-s001.zip › jcm-4541664-supplementary.pdf]

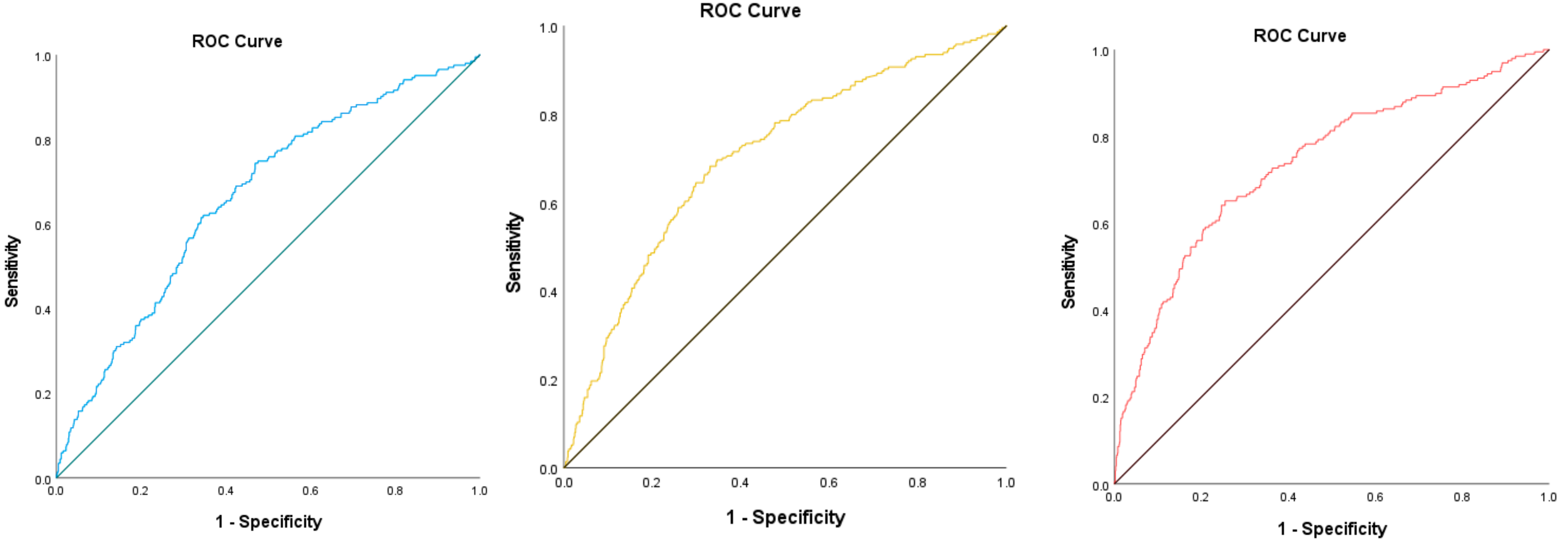

hs-CRP—TyG   WC: HT   hs- CRP—TyG +WC: HT

Figure S1. Receiver operating characteristic (ROC) curves for hs-CRP–TyG, WC: HT, and their combined model in predicting hypertension.

Hs-CRP—TyG

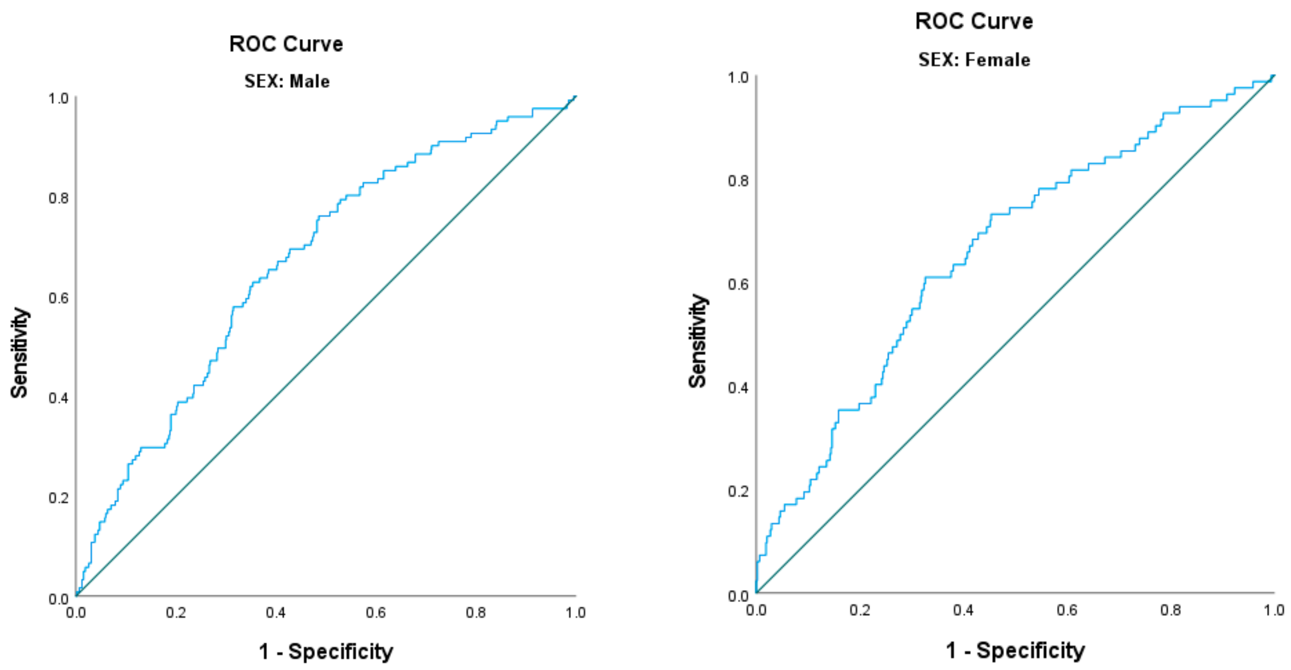

Figure S2. Receiver operating characteristic (ROC) curves for hs-CRP–TyG in male and female categories for prediction of hypertension.

WC: HT

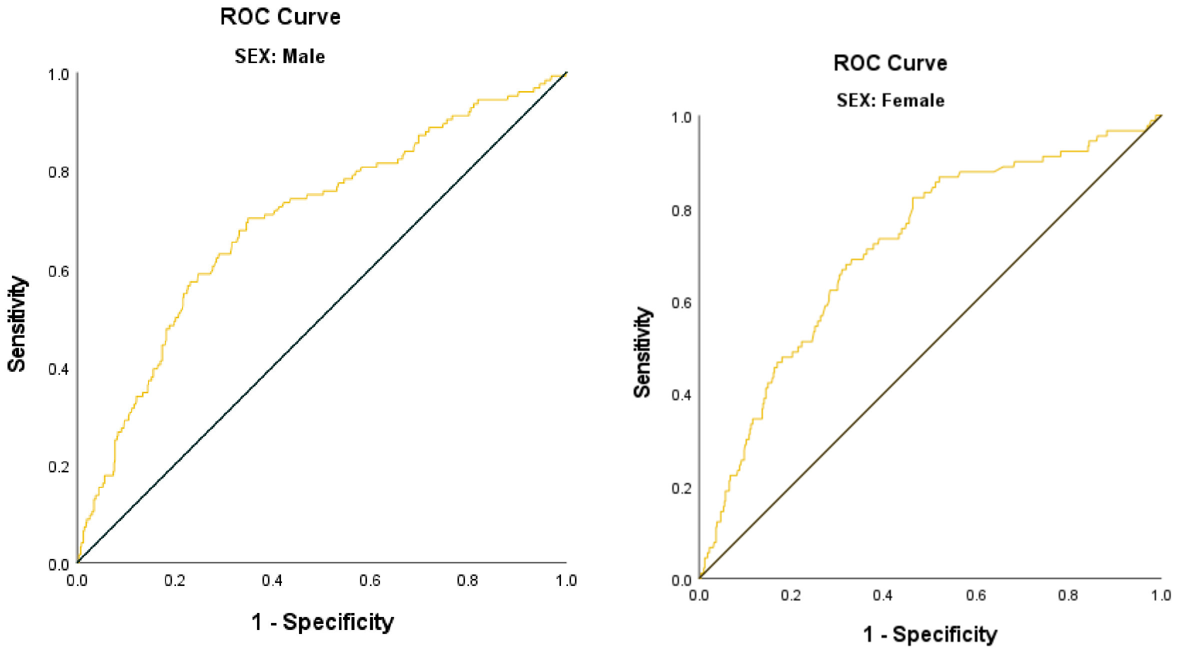

Figure S3. Receiver operating characteristic (ROC) curves for WC: HT model for prediction of hypertension by sex.

hs-CRP—TyG +WC: HT

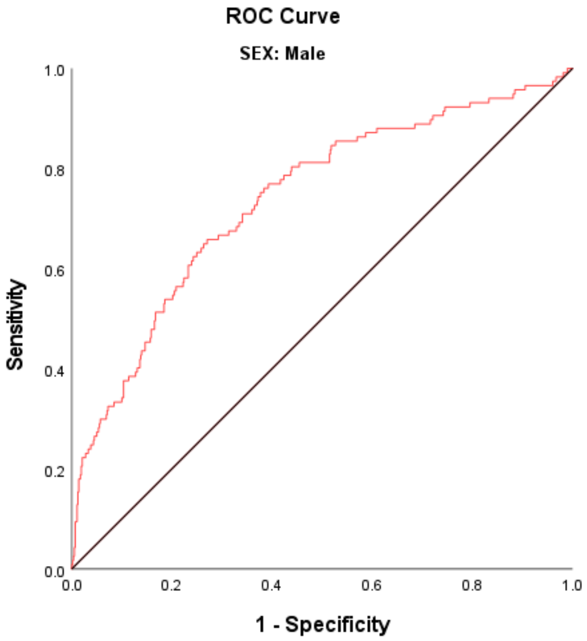

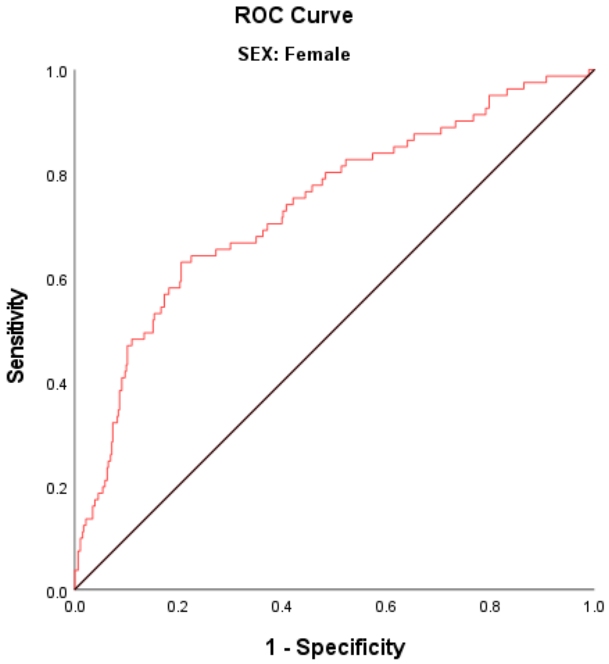

Figure S4. Receiver operating characteristic (ROC) curves for hs-CRP–TyG +WC: HT model for prediction of hypertension by sex.
